# Antagonistic Interaction of *Staphylococcus aureus* Toward *Candida glabrata* During *in vitro* Biofilm Formation Is Caused by an Apoptotic Mechanism

**DOI:** 10.3389/fmicb.2018.02031

**Published:** 2018-08-30

**Authors:** Omar Camarillo-Márquez, Itzel M. Córdova-Alcántara, Cesar H. Hernández-Rodríguez, Blanca E. García-Pérez, María A. Martínez-Rivera, Aida V. Rodríguez-Tovar

**Affiliations:** ^1^Laboratorio de Micología Médica, Departamento de Microbiología, Escuela Nacional de Ciencias Biológicas, Instituto Politécnico Nacional, Mexico City, Mexico; ^2^Laboratorio de Biología Molecular de Bacterias y Levaduras, Departamento de Microbiología, Escuela Nacional de Ciencias Biológicas, Instituto Politécnico Nacional, Mexico City, Mexico; ^3^Laboratorio de Microbiología General, Departamento de Microbiología, Escuela Nacional de Ciencias Biológicas, Instituto Politécnico Nacional, Mexico City, Mexico

**Keywords:** mixed biofilm, *Candida glabrata*, *Staphylococcus aureus*, antagonist interaction, cell-free bacterial supernatant, apoptosis

## Abstract

**Background:** Infections caused by *Candida* species and *Staphylococcus aureus* are associated with biofilm formation. *C. albicans–S. aureus* interactions are synergistic due to the significant increase in mixed biofilms and improved resistance to vancomycin of *S. aureus*. *C. glabrata* and *S. aureus* both are nosocomial pathogens that cause opportunistic infections in similar host niches. However, there is scarce information concerning the interaction between these last microorganisms.

**Results:** The relationship between *C. glabrata* and *S. aureus* was evaluated by estimating the viability of both microorganisms in co-culture of planktonic cells and in single and mixed biofilms. An antagonistic behavior of *S. aureus* and their cell-free bacterial supernatant (CFBS) toward *C. glabrata*, both in planktonic form and in biofilms, was demonstrated. Scanning electron microscopy (SEM), transmission electron microscopy (TEM), and confocal laser scanning microscopy (CLSM) images showed yeast cells surrounded by bacteria, alterations in intracytoplasmic membranes, and non-viable blastoconidia with intact cell walls. Concomitantly, *S. aureus* cells remained viable and unaltered. The antagonistic activity of *S. aureus* toward *C. glabrata* was not due to cell-to-cell contact but the presence of CFBS, which causes a significant decrement in yeast viability and the formation of numerous lipid droplets (LDs), reactive oxygen species (ROS) accumulation, as well as nuclear alterations, and DNA fragmentation indicating the induction of an apoptotic mechanism.

**Conclusion:** Our results demonstrate that the *S. aureus* CFBS causes cell death in *C. glabrata* by an apoptotic mechanism.

## Introduction

The National Institute of Health (NIH) reported that 80% of human infections are the result of biofilms established by pathogenic microorganisms (NIH guide, 1999). Candidiasis is a fungal infection caused by species of the genus *Candida*. Invasive candidiasis has been reported in approximately 75% of all systemic fungal infections and poses a serious threat to life, particularly in immunocompromised individuals, with mortality rates of 46–75% (Wilson et al., 2002). The most common species isolated are *Candida albicans*, *C. glabrata*, and *C. parapsilosis* (Diekema et al., 2012).

*Candida glabrata* is found as a commensal in the mucous membranes of healthy individuals. However, it can behave as an opportunistic pathogen, invades deeper tissues and gives rise to severe diseases when the host immune system is impaired. Additionally, this fungus possesses acquired resistance to azoles (Arendrup and Patterson, 2017).

Biofilms are microbial communities of surface-attached cells embedded in a self-produced extracellular polymeric matrix that constitutes a protected environment that is appropriate for growth and survival in hostile conditions (Costerton et al., 1999; Donlan and Costerton, 2002). Biofilm and planktonic cells exhibit distinctive biological properties, and a distinguishing feature of biofilms is their intrinsic tolerance to antimicrobials and immune attack (Hasan et al., 2009; d’Enfert and Janbon, 2016).

*Candida glabrata* has a great capability to form a biofilm on both infected tissues and inert surfaces, which can be considered a main virulence factor promoting infection and persistence in the host (Vijayalakshmi et al., 2016). *C. glabrata* is a producer of biofilm structures embedded in the extracellular matrix (ECM) (Kucharíková et al., 2015). *S. aureus* is a facultative anaerobic bacterium that is widely distributed through the world. In humans, this bacterium is found colonizing the skin and mucous membranes (Kloss, 1997; Otto, 2010). If these barriers are altered by trauma or surgery, *S. aureus* can access tissues, causing local infections. Due to its wide versatility, this bacterium can cause broad-spectrum infectious diseases such as bacteremia, osteomyelitis, toxic shock and infections, which involve the respiratory tract and urinary tract and central nervous system (Goetghebeur et al., 2007; Otto, 2010). *S. aureus* has an open pan-genome harboring a wide variety of virulence factors, such as adhesins, toxins and enzymes, and antimicrobial resistance genes (Gordon and Lowy, 2008; Bosi et al., 2016). The virulence and biofilm-forming capability of methicillin-resistant *S. aureus* (MRSA) have become a significant health problem (Goetghebeur et al., 2007; Silva-Santana et al., 2016). Biofilms can be formed on medical intravascular devices and serve as a source of nosocomial infections, which prolong hospitalization and increase mortality (Wisplinghoff et al., 2004; Silva-Santana et al., 2016).

In immunocompromised patients, *Candida* species and *S. aureus* can cause super-infections via the formation of mixed biofilms, for example, in the lungs of patients with cystic fibrosis (Bauernfeind et al., 1987). Interactions between species are organized and specific; for example, *Pseudomonas aeruginosa* adheres to the hyphae of *C. albicans*, but not to blastoconidia, inhibiting biofilm formation by *C. albicans.* Moreover, the interaction between *S. aureus* and *C. albicans* exhibits a synergistic activity, significantly improving biofilm formation and promoting the increase in *S. aureus* vancomycin resistance (Harriott and Noverr, 2009; Harriott and Noverr, 2010). Additionally, Kean et al. (2017) reported that during the association between these nosocomial pathogens, *S. aureus* takes advantage of the yeast biofilm architecture. To our knowledge, no *C. glabrata–S. aureus* mixed infections have been reported, although both species share the same habitat in humans. The aim of this work was to analyze the *in vitro* relationship between both microorganisms. The results revealed an antagonistic action of *S. aureus* over *C. glabrata*, as evaluated by their viability and architecture by SEM, TEM, and CLSM in co-culture of planktonic cells and during biofilm formation of *C. glabrata*, *S. aureus*, and *C. glabrata–S. aureus*. To describe the antagonistic mechanism, cell–cell contacts were analyzed using live and inactivated *S. aureus* cells, as well as treatment with CFBS; the latter showed a significant decrease in yeast viability and biofilm formation capability with LDs formation, accumulation of ROS and alterations in the cell membrane and chromatin condensation. Our findings demonstrated, that the antagonistic effect of *S. aureus* toward *C. glabrata* is due to an apoptotic mechanism.

## Materials and Methods

### Strains and Growth Conditions

*Candida glabrata* strain CBS138 (kindly donated by Professor Bernard Dujon from the Institute Pasteur, France) was stored in glycerol at -80°C and routinely cultivated in liquid YPD medium (1% yeast extract, 2% peptone, MCD Laboratorios, México for both and 2% glucose of J.T. Baker, Phillipsburg, NJ, United States) at 37°C under agitation (150 rpm). *S. aureus* strain ATCC 25923 was stored in glycerol at -80°C and cultivated in liquid BHI broth (MCD Lab) at 37°C. Both strains were sub-cultivated in solid YPD and BHI media with 2% agar.

### Effect of the First Colonizer on Mixed Biofilm Formation

Fungal and bacterial strains were incubated at 37°C in liquid YPD and BHI medium overnight, respectively, and harvested by centrifugation. The biomass was then washed twice with 5 mL of 1X sterile physiological solution PBS solution, pH 7.2. The inocula of both microorganisms were added to a suspension in Roswell Park Memorial Institute (RPMI) medium 1640 (Gibco, Gaithersburg, MD, United States) supplemented with 2% dextrose. *S. aureus* was adjusted to an OD at 610 nm (OD_610_) of 0.5 (approximately 1 × 10^8^ bacteria/mL), while *C. glabrata* was adjusted to 1 × 10^6^ yeast/mL utilizing a hemocytometer. Single and mixed biofilms were formed on 96-well flat-bottomed polystyrene plates (Nunc Roskilde, Denmark), and 200 μL of yeast and/or a bacterial suspension in RPMI 1640 was added to each well. The plates were incubated at 37°C for 4 h until the adherence stage. Then, the supernatant was eliminated to remove non-adhered cells, and 200 μL of fresh RPMI 1640 medium was added. Biofilm formation was evaluated at 0, 4, 8, 12, 24, 48, and 72 h at 37°C. The biofilm biomass was quantified according to the method described by Christensen et al. (1985) with modifications by Ramírez-Granillo et al. (2015) which employs crystal violet dye at 0.005% (final concentration). The excess dye was removed with distilled water followed by air-drying. Finally, the dye bound to the biofilm was solubilized with 200 μL of a solution of 33% (v/v) acetic acid (J.T. Baker) for 15 min. The absorbance of the solution was measured at a wavelength of 620 nm using a shaking Multiskan FC Microplate Reader (Thermo Fisher Scientific, Waltham, MA, United States). The OD values were proportional to the quantity of biofilm biomass. We changed the first colonizer microorganism during the adhesion phase to observe its influence on formation of the mixed biofilm. We used the same methodology described above with the following modifications: *C. glabrata* was inoculated and incubated for 4 h (adhesion stage); afterward, non-adherent cells were removed, the well containing the adherent cells was washed with 1X PBS, and *S. aureus* cells were inoculated and incubated at 37°C for 24 h. The same process was performed by first inoculating the bacteria and later the fungi. After incubation, the CFU/mL was counted. Each experiment was repeated three times with 12 technical replicates for each condition and controls.

### Viability of *C. glabrata* and *S. aureus* Single Cultures, Co-cultures and in a Mixed Biofilm

*Candida glabrata* and *S. aureus* were grown in single cultures and in co-culture. The inoculum was adjusted to 10^6^ yeast/mL and 10^8^ bacteria/mL, respectively, in RPMI 1640 medium at a final volume of 25 mL in a shake flask at 150 rpm, which was incubated for 0, 4, 8, 12, 24, and 48 h. The viability of the microorganisms was then determined, and decimal dilutions were performed and inoculated on BHI agar plates for *S. aureus* cultures and supplemented with 0.05 μg/mL amphotericin B (Sigma-Aldrich, St. Louis, MO, United States) for co-culture. The YPD plates for *C. glabrata* cultures were supplemented with 8 μg/mL vancomycin (Kener Laboratorios, México) for co-culture. The cultures were incubated at 37°C for different incubation times under agitation (150 rpm), and the CFU/mL were counted after incubation (Pesee et al., 2016). The assays were repeated in triplicate with two technical replicates for each condition and control.

The viabilities of *C. glabrata, S. aureus* single and mixed biofilms, and *C. glabrata* biofilm exposed to CFBS were also measured. The viability was determined at 0, 4, 8, 12, 24, and 48 h on 12-well microplates in which each well was washed twice with 1 mL of 1X PBS to remove non-adherent cells. Then, 1 mL of 1X PBS was added to each well to remove the formed biofilm using a sonicator (Gen Probe) for 1 min. The remaining cells were mixed by gently pipetting 10 times each, and the viability on biofilm cells was determined according to the CFU/mL as mentioned above.

### Structural Analysis by SEM and TEM

Individual and mixed biofilms were formed on 12-well microplates as described previously. The biofilms were incubated for 4, 12, 24, and 48 h. Subsequently, each well was washed (three times) with 1X PBS to remove non-adherent cells. The samples were fixed with 2.5% glutaraldehyde for 2 h and then post-fixed with 1% osmium tetroxide for 2 h. The samples were dehydrated by successive ethanol exposures from 10 to 90% for 10 min (each concentration). Final dehydration was achieved with two additional incubations in absolute ethanol for 20 min. The samples were coated with ionized gold for 40 s at 15,000 kV and 10 μA (Bozzola and Russell, 1999; Vázquez-Nin and Echeverría, 2000) and were observed using a SEM microscope (Quanta 3D FEG, FEI in Laboratory of Nanosciences, Micro and Nanotechnology Center IPN). Additionally, biofilm samples were processed for TEM as for SEM, but they were embedded in resin and allowed to polymerize overnight at 60°C. Semi-fine sectioning was performed with a Leica Ultracut UCT microtome (Wetzlar, Germany) and subjected to lead and uranyl solutions for contrast. Finally, the samples were mounted on slides for microscopic observation (JEOL Tokyo, Japan, in the Central Microscopy Laboratory, ENCB-IPN). Each experiment was repeated three times for each condition and controls.

### Observations of the Interactions and Viability of *C. glabrata and S. aureus* by CLSM

Single and mixed biofilms were developed as described previously in 12-well polystyrene plates containing a sterile coverslip (Velab, Mexico City, Mexico). Coverslips were recovered and placed in contact with a mixture of fluorochromes as follows: 1 g/L Calcofluor White (CW) (Sigma-Aldrich) for chitin and 10 mM FUN^®^-1 (Life Technologies, Gaithersburg MD, USA) for metabolic activity. Samples were observed under a CLSM (LSM5 Carl Zeiss, Germany) with the following filters: 355–433 nm (Calcofluor White), 480–530 nm (FUN^®^-1). Images were processed with Zeiss LSM Image Brower ver. 4.0 software (Carl Zeiss, Germany). Each experiment was repeated three times for each condition and controls.

### Effects of Cell-to-Cell Interactions of *S. aureus* on *C. glabrata* in Mixed Biofilms

A standardized overnight culture from BHI broth of *S. aureus* was harvested for 10 min at 13,000 × *g*. An aliquot of bacterial cells was heat-treated at 80°C for 30 min, and another aliquot was separately treated with methanol for 2 h. Subsequently, both inactivated bacterial cells were centrifuged for 10 min at 13,000 × *g* and adjusted to 10^8^ bacteria/mL in RPMI medium. The *C. glabrata* cells were adjusted to 10^6^ yeast/mL in RPMI medium and brought to a final volume of 200 μL with the same medium in 96-well polystyrene plates (Mowat et al., 2010). The single and mixed biofilms were evaluated after 24 h at 37°C and quantified according to Ramírez-Granillo et al. (2015) as previously described (three replicates). To test *S. aureus* inactivation, both samples were seeded in BHI medium and the absence of growth was detected. Each experiment was repeated three times with 12 technical replicates for each condition and controls.

### Effects of the Cell-Free Bacterial Supernatant (CFBS) of *S. aureus* on *C. glabrata* Biofilms

The CFBS was prepared after incubation of *S. aureus* at 37°C for 24 h in RPMI 1640 medium. Supernatants obtained by centrifugation were filtered through a 0.22 μm membrane. *C. glabrata* cells were adjusted to 1 × 10^6^ yeast/mL in RPMI 1640 medium and subjected to the CFBS at different dilutions (1:2, 1:4, 1:8, 1:16, and 1:32) in a final volume of 200 μL with the same medium in 96-well polystyrene plates (Saito and Ikeda, 2005; Holcombe et al., 2010). An aliquot of the CFBS was also heat-treated at 80°C for 30 min. The plates were incubated at 37°C for 4 h until the adherence stage. Then, the supernatant was eliminated to remove non-adhered cells, and 200 μL of fresh RPMI 1640 medium was added, the biofilm formation was evaluated for 24 h at 37°C, and was quantified as previously mentioned. Each experiment was repeated three times with 12 technical replicates for each condition and controls.

### Determination of the Viability of *C. glabrata* and *S. aureus* by the Tetrazolium Salt Assay

To evaluate the viability of *C. glabrata* and *S. aureus* single biofilms and *C. glabrata* single biofilms exposed to CFBS treatment, a colorimetric method based on the reduction of a tetrazolium salt 3-(4,5-dimethyl-2-thiazolyl)-2,5-diphenyl-2H-tetrazolium bromide (MTT) (Sigma-Aldrich) was performed as reported by Kairo et al. (1999) with modifications described by Walencka et al. (2005) as follows. After incubation in the wells of the microtiter plates for 0, 4, 8, 12, 24, and 48 h at 37°C, non-adherent cells were gently removed, and 100 μL of 1X PBS and 100 μL of 0.3% MTT were added and incubated for 2 h at 37°C in the dark. The MTT solution was removed from the wells, and 100 μL of DMSO (Sigma-Aldrich) and 25 μL of 0.1 M glycine buffer (pH 10.2) were immediately added to dissolve the formed formazan crystals in the wells. The incubation was carried out for 15 min at room temperature with slight shaking. The absorbance of the solution was measured at a wavelength of 450 nm using a shaking Multiskan FC Microplate Reader (Thermo Fisher Scientific). Each experiment was repeated three times with 12 technical replicates for each condition and controls.

### Detection of LDs in Cells of *C. glabrata* Exposed to CBFS

The effect of the CFBS of *S. aureus* on *C. glabrata* biofilms was also observed by the ultrastructure revealed by TEM with the above-mentioned protocol. The formation and accumulation of LDs was determined using the method described by Kimura et al. (2004) with minor modifications as follows. The *C. glabrata*–*S. aureus* mixed biofilm*, C. glabrata* single biofilm and *C. glabrata* single biofilm exposed to CFBS were used. The single and mixed biofilms developed as described above in 12-well polystyrene plates containing a sterile coverslip for 24 h at 37°C (Velab, Mexico City, Mexico) were subjected to 10 μL of Red Nile (0.1 mg/mL) (Sigma-Aldrich). After incubation for 10 min, the cells were observed under a CLSM (LSM5 Pascal, Carl Zeiss, Germany) at 450–520 nm. Images were processed with Zeiss LSM Image Brower ver. 4.0 software (Carl Zeiss, Germany). Each experiment was repeated three times for each condition and controls.

### Determination of ROS in *C. glabrata* Exposed to CFBS

To determine possible apoptotic markers induced in *C. glabrata* by exposure of *S. aureus* to CFBS, we analyzed single and mixed biofilms in 12-well polystyrene plates, which were developed as described above. The production and accumulation of ROS were analyzed using 2’,7’-dichlorofluorescin diacetate (DCFDA) reagent (Sigma-Aldrich) according to the method described by Zhu et al. (1994). We also investigated the cell membrane damage, by addition of 10 μL (100 μg/mL) of PI (Sigma-Aldrich) and incubation for 15 min at room temperature. The yeasts were then washed with 1X PBS for analysis with an epifluorescence microscope (Leica DMI 3000B; Leica Microsystems, Wetzlar, Germany) at 535–627 nm (Almshawit et al., 2014). Each experiment was repeated three times for each condition and controls.

### Terminal Deoxynucleotidyl Transferase-Mediated dUTP Nick End Labeling (TUNEL) Staining

The yeast DNA fragmentation was determined by TUNEL assay using the *in situ* cell death detection kit, fluorescein, from Roche Applied Science (Indianapolis, IN, United States). The biofilms were fixed with 4% (vol/vol) paraformaldehyde for 30 min at room temperature, washed three times with PBS 1X and the cell walls were digested with 24 μg/mL of Zymolyase 100 T (105 U/g, MP Biomedicals, Irvine, CA, United States) at 37°C for 60 min. The biofilms were rinsed with PBS, incubated in permeabilization solution [Triton X-100 at 0.1% (vol/vol) and sodium citrate at 0.1% (w/w)] for 2 min on ice and washed with PBS 1X. Next, the biofilms were subsequently incubated with 10 μL of TUNEL reaction mixture, containing terminal deoxynucleotidyl transferase and fluorescein isothiocyanate dUTP, for 60 min at 37°C (Madeo et al., 2002; Cabezón et al., 2016). Finally, the biofilms were rinsed three times with PBS. The observations were carried out under a CLSM (LSM5 Pascal, Carl Zeiss, Germany), excitation filter 450–490 nm and emission filter 520 nm.

### Specificity of the CFBS Inhibitory Effect Toward Different Yeasts

Following the basic procedures described in this paper, we test the inhibitory effect of CFBS toward several yeast species during biofilm formation. We use *Candida albicans*, *Cryptococcus neoformans*, *C. parapsilosis*, *C. krusei*, and *Saccharomyces cerevisiae* (Micología Médica lab ENCB-IPN collection). The biofilm quantification by performed with the Christensen method (Christensen et al., 1985). As control, was used the single biofilm of each yeast, and we analyzed the single biofilm exposed to CFBS as was described above.

### Statistical Analysis

The absorbance values of the single (*C. glabrata* or *S. aureus*) and mixed (*C. glabrata–S. aureus*) biofilms were compared using two-tailed analysis of variance (ANOVA). Student–Newman–Keuls and Holm–Sidak tests were used to determine significant differences employing SigmaPlot ver. 12.0 software (Systat Software Inc., San Jose, CA, United States) as appropriate.

## Results

### Biofilm Formation, Viability, and Effect of the First Colonizer on *C. glabrata–S. aureus* Interactions

**FIGURE 1:**
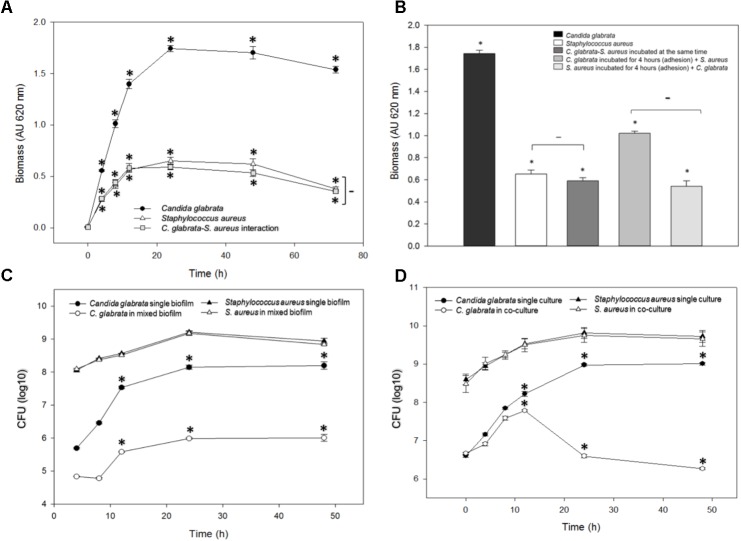
Quantification of the growth and viability of *C. glabrata* and *S. aureus* single and mixed biofilms. **(A)** Biofilm biomass was quantified indirectly by the crystal violet method after 0–48 h of incubation at 37°C for *C. glabrata*, *S. aureus* single biofilms and *C. glabrata*–*S. aureus* mixed biofilms in which a possible antagonistic interaction was observed due to the significant reduction (^∗^*p* < 0.050) of biomass compared with the single biofilm of *C. glabrata*. **(B)** Biofilm biomass was quantified after 24 h of incubation at 37°C for *C. glabrata*, *S. aureus* single biofilms and *C. glabrata*–*S. aureus* mixed biofilms. An evaluation of behavior according to the first colonizer of *S. aureus* and *C. glabrata* mixed biofilms was conducted. Fungus or bacterial inoculums were introduced as the first colonizer. **(C,D)** Viability was evaluated by the CFU assay for *C. glabrata* and *S. aureus* in single and mixed biofilm **(C)** and in single cultures, co-cultures with planktonic cells **(D)**, after 0–48 h of incubation at 37°C. In both cases, the viability of *C. glabrata* decreased significantly (^∗^*p* < 0.050) in co-culture and mixed biofilms with *S. aureus* compared with the single culture. In all cases, comparisons between absorbance (relative to biomass biofilm formation or CFU) revealed significant differences by the Student–Newman–Keuls or Holm–Sidak tests as appropriate, performing multicomparison of procedures. Values are representative of three experiments with 12 replicates each.

Biofilm formation was measured using the crystal violet method (see the section “Materials and Methods”) after different times of incubation at 37°C for *C. glabrata*, *S. aureus* single biofilms, and *C. glabrata–S. aureus* mixed biofilms; at 24 h, the biofilm production reaches its maximum value and stabilized (**Figure 1A**). In *C. glabrata–S. aureus* biofilms, the detected biomass (>0.4 AU) was significantly reduced (*p* < 0.001) compared with the single biofilm of *C. glabrata* (>1.6 AU), indicating a possible antagonistic behavior. There was no significant difference between the single *S. aureus* biofilm and *C. glabrata–S. aureus* mixed biofilms (**Figure 1A**).

With the aim to display the effect of the first colonizer in the biofilm formation, we compared the biofilms produced by *C. glabrata* or *S. aureus* by allowing adherence for 4 h. Subsequently, *S. aureus* or *C. glabrata* was added, respectively (**Figure 1B**). The effects exerted by modification of the first colonizer microorganism on the production of mixed biofilms demonstrated that when *C. glabrata* was the first colonizer, production of the mixed biofilm significantly increased compared with mixed biofilms developed from simultaneous inoculation. In contrast, there was no significant difference between *C. glabrata–S. aureus* mixed biofilms inoculated simultaneously and with *S. aureus* as the first colonizer (**Figure 1B**).

### Viability of Planktonic Cells of *C. glabrata* and *S. aureus* Single Cultures and Co-cultures

In the viability analysis during mixed biofilm formation, after 24 h of biofilm development with *S. aureus*, a significant reduction in *C. glabrata* counts (26.5%, *p* < 0.050) was noted in comparison to the CFU/mL of the single yeast biofilms. The counts of *S. aureus* did not decrease, and there were no significant differences in CFU/mL between *S. aureus* single culture biofilms and *C. glabrata–S. aureus* mixed biofilms (**Figure 1C**).

To quantitatively analyze the inhibitory effect of *S. aureus* on *C. glabrata* viability, the colony-forming-units CFU assay was performed for *C. glabrata* and *S. aureus* in single cultures and in co-culture after 0, 4, 8, 12, 24, and 48 h of incubation at 37°C. After 24 h of incubation, the viability of *C. glabrata* decreased significantly (27.20%, *p* < 0.050) in co-culture with *S. aureus* compared with single culture, and after 48 h, there was a decrease in yeast viability of 30% (*p* < 0.050). However, *S. aureus* viability was not influenced in co-culture with *C. glabrata* compared with their single cultures (**Figure 1D**).

### Ultrastructure of Single and Mixed Biofilms Formed by *C. glabrata* and *S. aureus* by SEM and TEM

**FIGURE 2:**
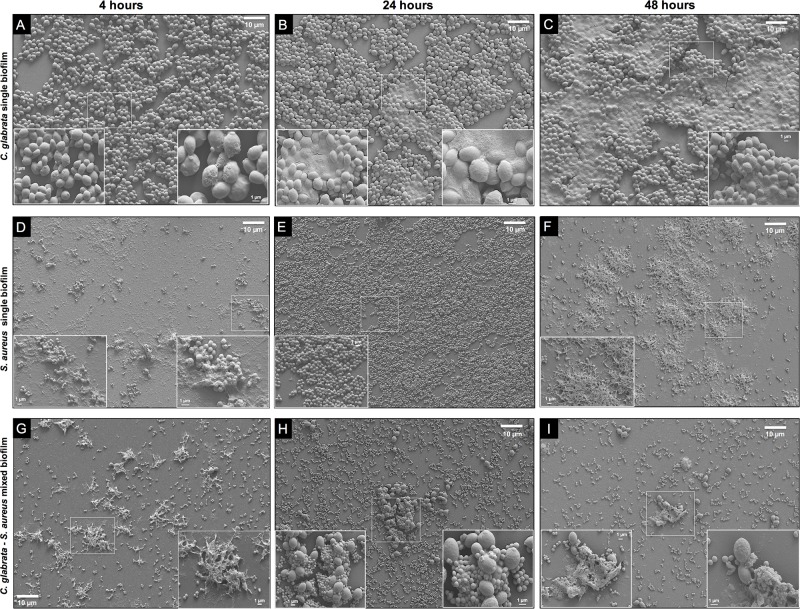
Scanning Electron Microscopy (SEM) of *C. glabrata* and *S. aureus* single and *C. glabrata*–*S. aureus* mixed biofilms. *C. glabrata* single biofilm **(A)** 4 h, **(B)** 24 h, and **(C)** 48 h. *S. aureus* single biofilm **(D)** 4 h, **(E)** 24 h, and **(F)** 48 h. *C. glabrata–S. aureus* mixed biofilms **(G–I)** revealed structural alterations in the fungus–bacterium interaction compared with single biofilms **(G)** 4 h, **(H)** 24 h, and **(I)** 48 h. Biofilms that developed in 12-well polystyrene plates in RPMI medium at 37°C. The white box insert shows a higher magnification detail. Images are representative of three experiments each.

Micrographs obtained by SEM of single biofilms of *C. glabrata*, *S. aureus* and mixed biofilms were observed at 4, 8, 12, 24, and 48 h as described previously. Images of *C. glabrata* single biofilms at 4 h, the adhesion stage, with cell co-aggregation, cell proliferation, budding yeasts, and the production of EPS were obtained (**Figure 2A**). At 24 h, the maturation stage was observed, with a marked increase in cell proliferation and ECM surrounding the cells. The biofilm displayed a large structural arrangement of cell co-aggregation embedded in the ECM and channel formation (**Figure 2B**). At 48 h, we could observe the phase of disassembly by cellular detachment, empty biofilm spaces, and large amounts of ECM (**Figure 2C**). *S. aureus* biofilm images revealed typical characteristics of bacterial biofilms with minimal differences compared with the stages of yeast biofilm (**Figures 2D–F**). At 4 h, the initial stage, co-aggregation of bacterial cells was observed, with high production of EPS, which allows adhesion to the biofilm support surface (**Figure 2D**). At 24 h, maximum biofilm formation was observed, and the bacteria were organized in microcolonies with three-dimensional structures (3D) and a rough topography due to the abundant production of ECM that occurs in the mature biofilm. The formation of extensions known as polymeric bridges was also observed, and the microcolonies formed channels to allow fluids to move outside the microcolonies (**Figure 2E**). The disassembly stage was observed after 48 h of incubation (**Figure 2F**) and was characterized by cell detachment, empty spaces in the biofilm and large amounts of ECM. Regarding the mixed biofilm with the antagonistic relationship of *S. aureus* toward *C. glabrata* observed by SEM (**Figures 2G–I**), the early stage of mixed biofilm formation (4 h) showed EPS production with embedded yeast and several cocci surrounding a few blastoconidia (**Figure 2G**). Biofilms that formed at 24 h had an evident diminution of embedded blastoconidia in a thin layer of ECM compared with bacterial cells (**Figure 2H**). During the disassembly stage (48 h), we observed an important reduction of bacterial cells and fungal cells, with a few blastoconidia embedded in the ECM, surrounded by bacterial cells (**Figure 2I**).

**FIGURE 3:**
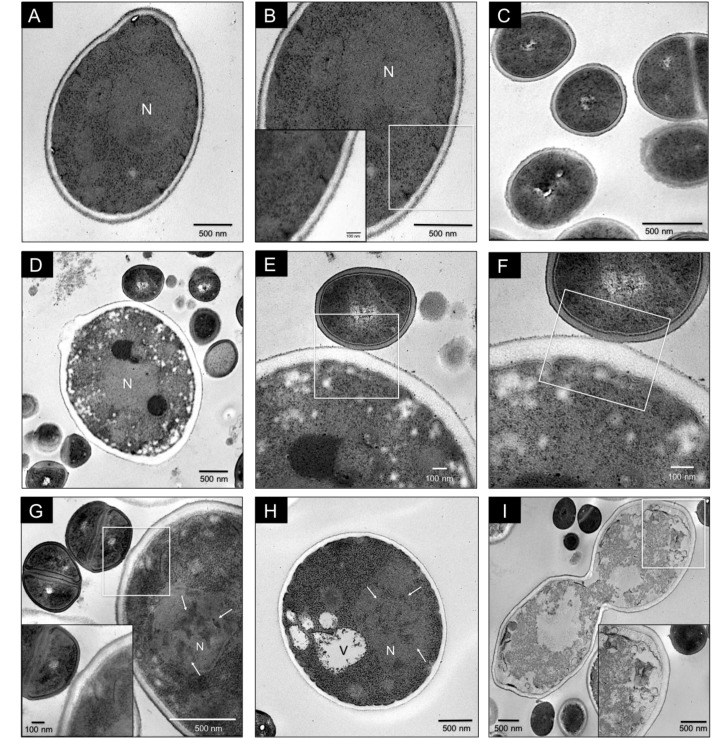
Transmission electron microscopy (TEM) of *C. glabrata* and *S. aureus* single and mixed biofilms. *C. glabrata* single biofilm **(A,B)**. Different magnifications of blastoconidia with a well-defined structural organization, delimited intracytoplasmic organelles, single nucleus (N), cytoplasm with homogeneous electro-density and cellular bud scar at 24 h. **(C)** *S. aureus* single biofilm at 24 h. **(D–F)** *C. glabrata–S. aureus* mixed biofilms at 12 h, showing specific adherence of the cocci to the yeast wall and discontinuity of the cell membrane. **(G–I)** *C. glabrata–S. aureus* mixed biofilms at 24 h. **(G)** Cocci attached to the fungus wall; blastoconidia with severe cell membrane alterations, organelle disorganization, chromatin condensation (white arrows), and vacuole formation (V). The white box inset shows a higher magnification detail. **(H)** Blastoconidia with chromatin condensation (white arrows). **(I)** Loss of cytoplasmic homogeneity (electro-density) with organelle degradation and yeast cell death. The white box inset shows a higher magnification detail. Samples were recovered from 12-well polystyrene plates in RPMI medium at 37°C. The white box insert shows a higher magnification detail. Images are representative of three experiments each.

Antagonistic interaction in *C. glabrata–S. aureus* mixed biofilms was also shown by TEM. Images of *C. glabrata* in single biofilms (**Figures 3A,B**) at 24 h exhibited a blastoconidium with well-defined membranous structures, a single nucleus, a typical range of delimited intracytoplasmic organelles, and a well-defined bud scar. Furthermore, the cytoplasm displayed a homogeneous electro-density and intact membranes. *S. aureus* single biofilm TEM image (**Figure 3C**) showing some cocci with well-defined cell walls and a homogeneous electro-dense cytoplasm. Images of mixed biofilms formed by *C. glabrata–S. aureus* at 12 h (**Figures 3D–F**) show adherence of the cocci to the yeast wall, with alterations in the continuity of the cell membrane (noteworthy in the inset). Additionally, after 24 h, this antagonistic activity of *S. aureus* toward *C. glabrata* is notable based on the yeast ultrastructure (**Figures 3G,H**), but with cocci without alterations. We found blastoconidia with severe alterations in the cell membrane as equivalent discontinuities, together with the disappearance of organelle organization. The fungal cells had less electro-dense cytoplasm with some intact cells walls. Vacuole formation, evident chromatin condensation and, ultimately, cellular lysis are shown in **Figure 3I**, but uninjured bacterial cells can be observed.

### Evaluation of the Viability of *C. glabrata–S. aureus* Mixed Biofilms by CLSM

**FIGURE 4:**
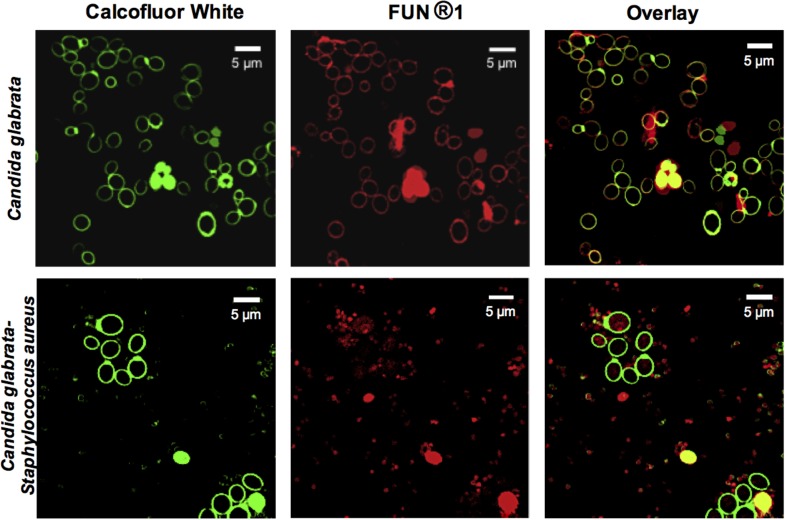
Confocal Laser Scanning Microscopy (CLSM) images of *C. glabrata* and *C. glabrata–S. aureus* mixed biofilms. *C. glabrata* single and mixed with *S. aureus* biofilms. The CLSM image shows a diminution of *C. glabrata* metabolic viability (FUN^®^-1, red mark) in the mixed biofilm compared with the *C. glabrata* single biofilm, as evidenced by Calcofluor White staining of chitin (green halo). Biofilm development was performed in 12-well polystyrene plates with cover-slips and incubation at 24 h with RPMI medium at 37°C. Images are representative of three experiments each.

In addition, to demonstrate the viability of *C. glabrata* in single and mixed biofilms, the biofilms were analyzed using different fluorochromes (**Figure 4**). The fluorochrome FUN^®^-1 (red halo) was used to observe metabolic activity, and Calcofluor White (green halo) was used to identify chitin. The co-localization of chitin and metabolic activity, was observed by the overlap of the signals emitted by the fluorochromes. The interaction of *C. glabrata–S. aureus* led to the non-viability of *C. glabrata*, as evidenced by their metabolic activity. CLSM detection of this last fluorochrome confirmed that, in general, *S. aureus* had antagonistic effects toward *C. glabrata* in the biofilm, in comparison to their single biofilms, confirming the data from the CFU assay.

### Effects of Cell-to-Cell Interactions Between *C. glabrata – S. aureus* in Mixed Biofilms

**FIGURE 5:**
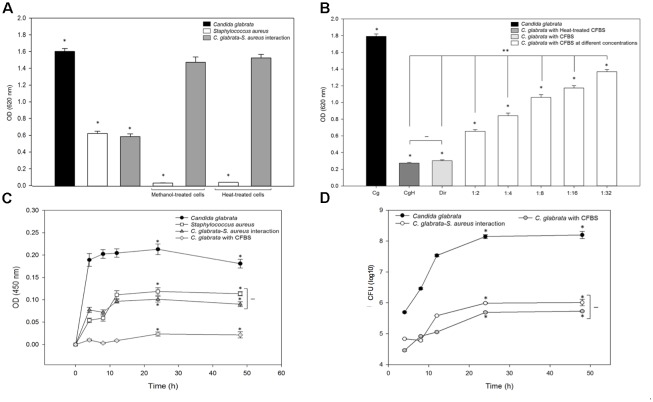
Antagonistic effect of *S. aureus*-inactivated cells and cell-free bacterial supernatant (CFBS) on *C. glabrata* viability during biofilm formation. **(A)** Biofilms formed by cell–cell interactions were quantified indirectly by the crystal violet method at 37°C for 24 h with inactivated cells (treated with heat and methanol, separately) of *S. aureus* mixed with *C. glabrata*; there was no significant reduction in *C. glabrata* biofilm production following exposure to inactivated *S. aureus.* **(B)** Evaluation of the effect of CFBS on *C. glabrata* during biofilm formation by indirect quantification by the crystal violet method at 37°C for 24 h. Fresh and heat-treated CFBS had a significant inhibitory effect on the *C. glabrata* biofilm, compared with the *C. glabrata* single biofilm in the presence of all tested CFBS amount. The viability of *C. glabrata* decreased significantly (^∗^*p* < 0.050) in the presence of CFBS heat-treated (CgH), direct CFBS (Dir), and with CFBS at different concentration compared with the single culture. It was existed significantly difference (^∗∗^*p* < 0.050) between CFBS at different concentrations, CgH and Dir. But did not exist significantly difference (–) between CgH and Dir. **(C)** Metabolic activity was determined using the MTT assay after 0–48 h of incubation at 37°C for *C. glabrata*, *C. glabrata* with CFBS and *S. aureus* single and mixed biofilms. **(D)** Viability of the *C. glabrata* biofilm exposed to CFBS was assessed by the CFU assay after 4–48 h of incubation at 37°C. The viability of *C. glabrata* decreased significantly (^∗^*p* < 0.050) in the presence of CFBS compared with the single culture. Significant differences were determined using the Holm–Sidak and Student–Newman–Keuls tests as appropriate, performing multicomparison of procedures. Values are representative of two experiments with three replicates each.

To determine if the inhibitory effect was caused by cell-to-cell contact, bacterial cells were killed (treated with heat or methanol) during *C. glabrata* biofilm formation. The inactivated bacterial cells were mixed with *C. glabrata* and incubated for 24 h at 37°C. The results showed that there were no significant differences between the *C. glabrata* biofilm and the biofilm formed by *C. glabrata–S. aureus* when the bacterial cells were inactivated by heat or methanol, as shown in **Figure 5A**.

### Effects of the Cell-Free Bacterial Supernatant (CFBS) of *S. aureus* on *C. glabrata* Biofilms

To demonstrate whether the antagonistic relationship between *C. glabrata* and *S. aureus* was influenced by the release of secreted bacterial extracellular molecules, we tested the effect of the CFBS on *C. glabrata* biofilms (**Figure 5B**). The capacity of *C. glabrata* to form a biofilm was affected following exposure to CFBS in a dose-dependent manner. When the yeast was exposed directly to CFBS, the *C. glabrata* biofilm was significantly reduced (83.24%, *p* < 0.050) in comparison with the control. Exposure of *C. glabrata* to different amounts of CFBS also resulted in a reduction of the biofilm biomass from 24 to 63% at dilutions of 1:2 (63.69%, *p* < 0.050), 1:4 (53.08%, *p* < 0.050), 1:8 (40.79%, *p* < 0.050), 1:16 (34.64%, *p* < 0.050) and 1:32 (24.03%, *p* < 0.050) (**Figure 5B**). Heat treatment of CFBS did not eliminate the inhibitory effect on the biofilm formation capacity (**Figure 5B**).

### Determination of *C. glabrata* Viability by the Tetrazolium Salt (MTT) Assay Following Exposure to CFBS

We further used the MTT methodology to evaluate the metabolic activity of *C. glabrata, S. aureus* single and mixed biofilms and *C. glabrata* exposed to CFBS. Our results showed that *C. glabrata* biofilms had higher metabolic activity (OD_450__nm_) of 0.2 AU, while *S. aureus* showed a value of 0.11 AU. In the fungus–bacterium interaction, there was no significant difference compared with *S. aureus* alone based on equivalent metabolic activities. However, the presence of CFBS dramatically inhibited metabolic activity in *C. glabrata* biofilms (88.92 reduction of the percentage at 24 h, *p* < 0.05) compared with the mixed and *C. glabrata* biofilms. All metabolic activities were measured in single and mixed biofilms after 0, 4, 8, 12, 24, and 48 h at 37°C (**Figure 5C**). The results were corroborated by measuring the CFU/mL, and a significant difference was observed between single *C. glabrata* biofilms and *C. glabrata* biofilms exposed to CFBS (**Figure 5D**).

### Ultrastructure of *C. glabrata* Biofilms Exposed to CFBS by TEM

**FIGURE 6:**
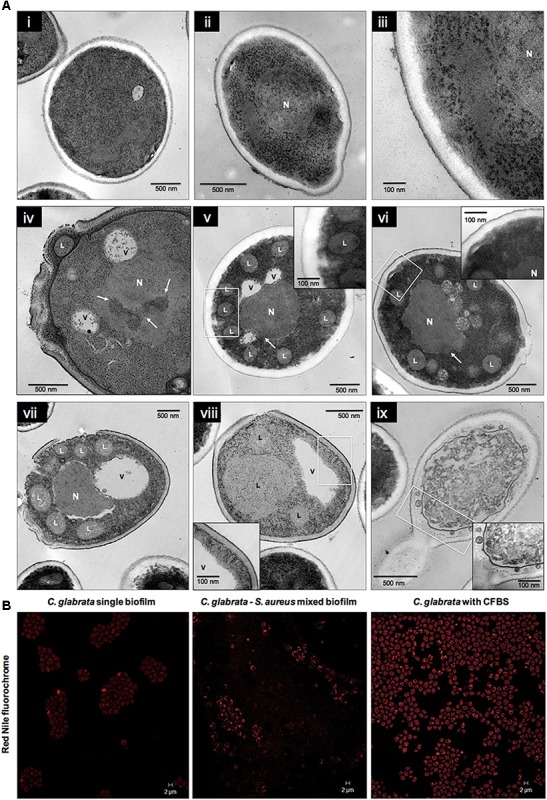
Ultrastructural alterations in *Candida glabrata* cells induced by CFBS after 24 h of incubation. **(A)** TEM micrographs of *C. glabrata* single biofilm and in the presence of CFBS. *C. glabrata* single biofilm. **(A, i–iii)** Different magnifications of blastoconidium with typical characteristics of yeast wall, intact organelles, single nucleus (N), cytoplasm with a homogeneous electro-density and a cellular bud scar at 24 h. **(A, iv–ix)** Blastoconidia at 24 h with acute alterations of the cell membrane and fungal cell wall, and the formation of several presumptive LDs, vacuoles (V), chromatin condensation (white arrows), and cell membrane invagination. Fungal wall damage and even rupture, a loss of electro-density homogeneity in the cytoplasm, organelle disorganization and degradation, and plausible cell death were observed. The white box inset shows a higher magnification. **(B)** CLSM images of stained LDs in *C. glabrata* single biofilms, mixed biofilms and in the presence of CFBS. LDs were stained with the Nile Red fluorochrome, as evidenced in yeasts, which showed an accumulation of these LDs in mixed biofilms and when the yeast were exposed to CFBS.

Transmission electron microscopy analysis at 24 h of the *C. glabrata* biofilm exposed to CFBS showed the apparent presence of abundant LDs, vacuole formation, changes in cell membrane and fungal wall continuity, and changes in the nuclear membrane in which chromatin condensation and empty space between the nucleus and cytoplasm were observed (**Figure 6A** iv–ix) compared with *C. glabrata* single biofilms (**Figure 6A** i–iii). These results are likely to be characteristic of programmed cell death. To determine the nature of the presumptive LDs, *C. glabrata* biofilms were stained with Nile Red fluorochrome, which revealed a large accumulation of LDs in mixed biofilms and when the yeasts were exposed to CFBS (**Figure 6B**), compared with *C. glabrata* single biofilms.

### Determination of ROS in *C. glabrata* Exposed to CFBS

**FIGURE 7:**
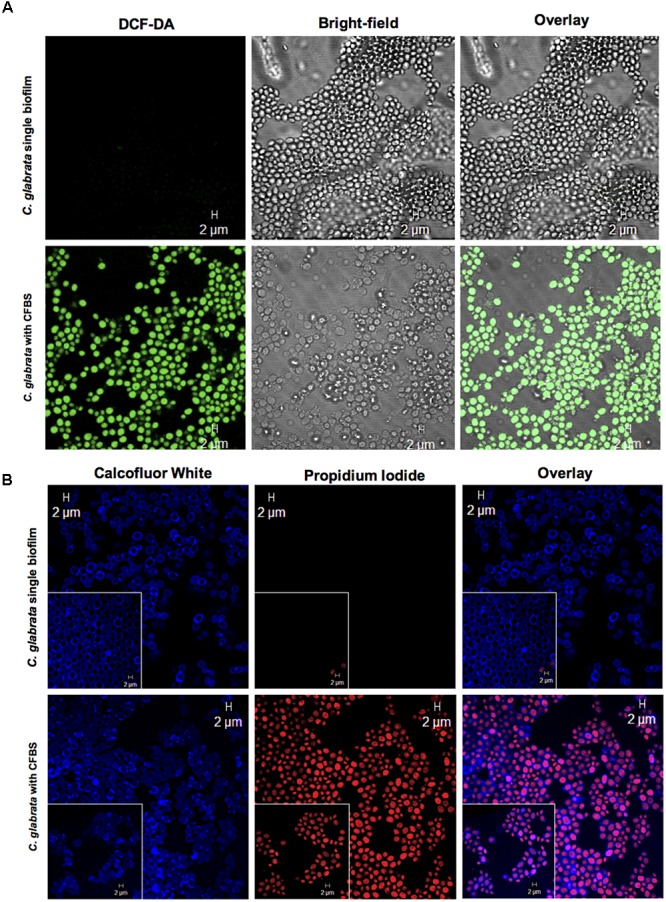
Apoptotic marker detection in *C. glabrata* with CFBS by CLSM. **(A)** ROS detection in *C. glabrata* single biofilms and with CFBS. ROS-positive cells were identified by green fluorescence only in *C. glabrata* with CFBS. *C. glabrata* single biofilms did not exhibit any fluorescence. **(B)** Cell membrane damage detection PI-positive cells were identified by red fluorescence. The images show yeast cell membrane injury caused by CFBS compared with the *C. glabrata* single biofilm, as evidenced by Calcofluor White, which showed an intact fungal cell wall. The white box inset shows a higher magnification detail. Cell membrane damage in *C. glabrata* was also examined after exposure to CFBS **(B)**. We did not observe any fluorescence signal in single *C. glabrata* biofilms that were not exposed to CFBS.

To assess whether *S. aureus* and their CFBS could induce ROS accumulation, we used the 2,7-dichlorodihydrofluorescein diacetate (DCFH-DA) reagent to identify these metabolites. During single *C. glabrata* biofilm formation, *S. aureus* (**Supplementary Figure S1**) and their CFBS induced a higher level of ROS accumulation than the single yeast biofilm after 24 h at 37°C (**Figure 7A**).

### Determination of DNA Fragmentation in *C. glabrata* After CFBS Exposure by TUNEL Assay

**FIGURE 8:**
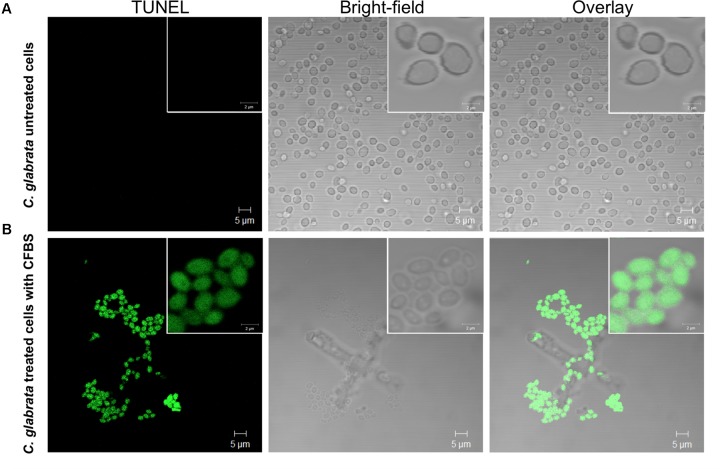
Determination of death of *C. glabrata* by TUNEL method. TUNEL-positive color development (green) indicating DNA strand breakage was observed in *C. glabrata* exposed to CFBS produced by *S. aureus*. **(A)** *C. glabrata* untreated cells, and **(B)** *C. glabrata* CFBS treated cells.

All *C. glabrata* cells treated with CFBS showed TUNEL-positive nuclei while, none of the untreated *C. glabrata* control (**Figure 8**). These results, demonstrated, that the antibiosis effect of CFBS toward *C. glabrata* is due to an apoptotic mechanism.

### The CFBS Antagonic Effect Is Specific to Non-albicans Candida

**FIGURE 9:**
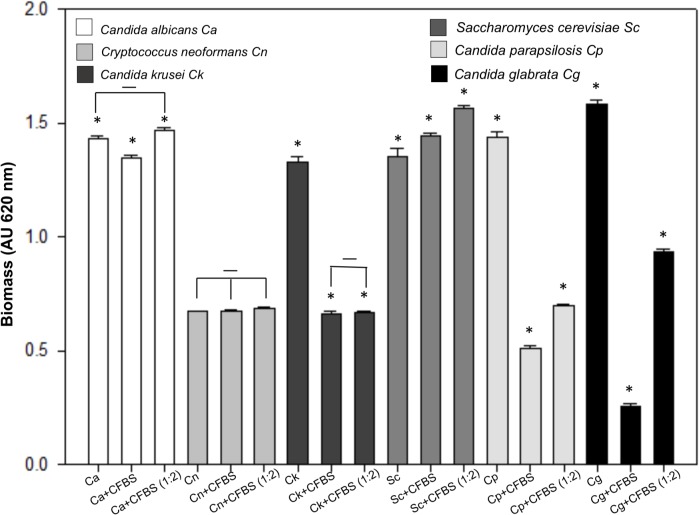
Quantification of the growth of several yeast species single biofilm exposed to CFBS. Biofilm biomass was quantified indirectly by the crystal violet method after 24 h of incubation at 37°C of *C. albicans, C. neoformans, C. krusei, S. cerevisiae, C parapsilosis*, and *C. glabrata*. The single biofilms non-albicans Candida exposed to CFBS show a possible antagonistic interaction due to the significant reduction (^∗^*p* < 0.050) of biomass between untreated and treated yeast cells single biofilm. While *C. albicans, C. neoformans*, and *S. cerevisiae* not showed significant biomass reduction.

This experiment shown an interesting behavior. We found that the CFBS is able to inhibit the single biofilm formation of *C. glabrata, C. parapsilosis*, and *C. krusei*, but not *C. albicans, C. neoformans* and *S. cerevisiae* (**Figure 9**).

## Discussion

Microbes rarely exist as single-species in planktonic forms; the majority is found thriving in complex polymicrobial biofilms (Peters et al., 2012). Interactions within these biofilms can be mutualistic, commensalistic, or antagonistic (Peters et al., 2012; Cabezón et al., 2016). Fungal–bacterial interactions often result in changes in the pathogenicity, metabolic functioning, and expression of virulence factors of one or both participants (Frey-Klett et al., 2011; Willems et al., 2016; Costa-Orlandi et al., 2017). These interactions are important in a variety of disease states and niches, including infections of the respiratory system, the formation of dental plaque, invasive disease, skin and mucosal infections, bloodstream infections and intra-abdominal infections (Frey-Klett et al., 2011; Scherlach et al., 2013; Dixon and Hall, 2015; Lilly et al., 2018). In this study, we report, to our knowledge for the first time *in vitro*, an antagonistic effect of *S. aureus* over *C. glabrata* toward mixed biofilms, planktonic co-cultures and with CFBS using different methodologies. Our results indicate that *S. aureus* inhibits the development of *C. glabrata* by decreasing its viability, altering its morphology and even causing lysis and cell death. First, the antagonist behavior of the interaction between *C. glabrata* and *S. aureus* was established during *in vitro* biofilm formation, during which we observed that the bacterium could radically decrease the amount of yeast biofilm biomass (**Figures 1A,B**). Using a CFU assay in co-culture (**Figure 1C**) and mixed biofilm (**Figure 1D**), we found that the addition of *C. glabrata* and its adherence may have provided a greater opportunity for biofilm formation, even when it was in contact with *S. aureus;* however, the biofilm biomass formed by the bacterium was not significantly different compared with the controls (**Figures 1A,B**). To observe this relationship in more detail, we used SEM. We observed the typical structural features defining the different stages of biofilm formation (**Figures 2A–F**). In general, *C. glabrata* developed compact biofilms formed exclusively of blastoconidia embedded within an ECM as reported previously (Seneviratne et al., 2009). The *C. glabrata* biofilm matrix includes proteins and carbohydrates, among which β-1,3-glucans are the most important (Nett et al., 2007; Seneviratne et al., 2009). This latter component forms a dense wall that prevents drug and antifungal diffusion. The matrix is shown in **Figures 2B,C**. The *C. glabrata* biofilm also presents tolerance to azoles and polyenes but is susceptible to echinocandins and lipid formulations of amphotericin B (Seneviratne et al., 2009; Silva et al., 2010; d’Enfert and Janbon, 2016; Rodrigues and Henriques, 2017). The capacity for biofilm formation of *S. aureus* (**Figures 2D,E**) is considered a main virulence factor that directly influences the survival and persistence of bacteria in the environment as well as in the host (Silva et al., 2010; Rodrigues and Henriques, 2017; Torlak et al., 2017). The antagonism of *S. aureus* toward *C. glabrata* was also observed by SEM, which revealed an evident reduction of blastoconidia in the different microscopic fields (**Figures 2G–I**). This finding was directly related to the viability count results (CFU/mL) obtained for the mixed biofilms described above (**Figure 1D**). During the maturation stage in the mixed biofilm, we observed *S. aureus* attached to the surface of *C. glabrata* (**Figure 2H**). Ikeda et al. (2007) reported a similar behavior of *C. neoformans* and *S. aureus* in which the bacterium also had a high affinity for the blastoconidial cell wall, and they concluded that adherence was mediated by the action of the enzyme triosephosphate isomerase, which interacts with fungal carbohydrates from the cryptococcal capsule. The authors considered the damage to the fungus cell wall to be the cause of the antagonistic effect of *S. aureus*. An antibiosis effect of *S. aureus* toward the filamentous fungus *A. fumigatus* has also been described in mixed biofilms in which minimal production of the biomass biofilm, abortive hyphae, limited hyphal growth, alterations of conidial shape and deformation of fungal structures were observed (Ramírez-Granillo et al., 2015). Bandara et al. (2010) demonstrated by SEM that *P. aeruginosa* and *Candida* spp. in a dual species environment mutually suppressed biofilm development, and the biofilms of mature monospecies showed a characteristically thick layered structure. However, in contrast, dual species biofilms consisted of less dense growth of *Candida* spp. and *P. aeruginosa* (Kerr, 1994).

We use TEM to corroborate the antagonistic effect. In mixed biofilms, we observed several alterations of the fungal structures, including disorganization of the cell membrane, cell wall, and intracytoplasmic organelles and a less electro-dense cytoplasm, and when the cocci were in contact with blastoconidia, we observed a slightly electro-dense material between both (**Figure 3**). We did not observe any modifications of the bacterial morphology, as shown in **Figure 3C**, we suggest that this material could be a mannose-binding lectin (MBL) on the bacterial cell surface. However, its nature is still unknown (Neth et al., 2002; Bandara et al., 2010) that is likely to induce the high adherence capacity of *S. aureus* to *C. glabrata*. Additionally, as shown in **Figures 3G,H**, we observed chromatin condensation in the blastoconidia, which is a typical characteristic of a type of programmed cell death (Cabezón et al., 2016). To better understand the previous results, we determined the cell viability during the interactions of *C. glabrata–S. aureus* by CLSM using different fluorochromes, and we also demonstrated an antagonistic effect of *S. aureus* toward *C. glabrata*. FUN^®^-1 allowed us to observe viable *C. glabrata* cells during the interaction, while calcofluor white was applied to identify chitin in the yeast cell wall, regardless of whether they were alive or dead. These fluorochromes revealed the presence of non-viable yeast during this interaction (**Figure 4**). These results suggest that the antagonistic effect toward the yeast viability is due to the presence of *S. aureus.*

To assess whether the inhibitory effect was due to cell-to-cell contact or whether *S. aureus* was capable of secreting a molecule with antifungal properties, we used inactivated *S. aureus* cells and CFBS according to different researchers (Neth et al., 2002; Ikeda et al., 2007). We demonstrated that cell-to-cell contact was not necessary for fungal killing because inactivated cells did not have any effect on the *C. glabrata* biofilm (**Figure 5A**); however, when the yeast were treated with CFBS, we observed a clear inhibitory effect, suggesting the presence of an inhibitory substance that shows heat-resistance (**Figure 5B**). Colorimetric assays are commonly used in studies of the development and susceptibility of biofilms to antifungal drugs (Seneviratne et al., 2009). To quantify the metabolic activity of *C. glabrata* following exposure to CFBS, we used the MTT assay (Hawser and Douglas, 1994). We observed a decrease in metabolic activity of *C. glabrata* (**Figure 5**).

Transmission electron microscopy micrographs also confirmed the presence of damage inside the blastoconidia exposed to CFBS (**Figure 6A** iv–ix), including intracytoplasmic disorganization, cell membrane discontinuity, vacuoles, and chromatin condensation. However, in addition to the injury induced in the structure of yeast by the supernatant, we observed the formation of presumptive LDs, and the nature of these structures was confirmed by Red Nile fluorochrome staining (**Figure 6B**). LDs are dynamic intracellular organelles that contain neutral lipids as their main constituents, including TAGs and SEs. LDs participate in multiple cellular functions, such as membrane trafficking, phospholipid recycling, intracellular protein metabolism and cell signaling (Chang et al., 2015). Chang et al. (2015) performed assays using an endolichenic fungus, *Phaeosphaeria* sp., which biosynthesizes PQs to demonstrate the probable function of LDs during toxin resistance in fungi. Their results suggested that the LDs could trap endogenous or external lipophilic phototoxins, representing a resistance mechanism that protects against toxins in both the producer and the recipient. They concluded that fungal LDs play crucial roles in drug resistance and adaptation to stress (Chang et al., 2015). Based on these results compared with our findings, we suggest that the great accumulation of LDs within *C. glabrata* is probably a response to a substance in the CFBS produced by *S. aureus*.

Yeast apoptosis has been considered a mechanism of adaptation in response to adverse environmental conditions (Skulachev, 2002) and was first described in a cdc48 mutant (Madeo et al., 1997). Elevated levels of ROS occur in response to different types of external stimuli that are detrimental to the cell, such as high doses of hydrogen peroxide, acetic acid, sodium chloride or other types of stressors such as chronological and replicative aging. The ROS are delivered from the cytosol to the nucleus, where they are involved in DNA fragmentation (Madeo et al., 1997; Skulachev, 2002; Phillips et al., 2003). To corroborate whether the chromatin condensation and cell death of *C. glabrata* observed in the TEM micrographs were mediated by an apoptotic process, we used typical apoptotic markers and observed the results by CLSM. ROS accumulation and staining with PI revealed a great accumulation of ROS in the blastoconidia (**Figure 7A**) and damage at the cell membrane level, as observed by entry of the PI fluorochrome into the yeast cells (**Figure 7B**). The TUNEL method is a fast and sensitive way to visualize the DNA fragmentation cells (Madeo et al., 1997; Skulachev, 2002; Phillips et al., 2003; Cabezón et al., 2016). We consider fungal apoptosis in *C. glabrata* cells caused by CFBS exposition, due to the positive result of the terminal deoxynucleotidyl transferase-mediated dUTP nick end labeling assay suggest that the death of *C. glabrata* was by DNA fragmentation (**Figures 8A,B**).

After exposition to CFBS produced by *S aureus*, the *C. glabrata* cells showed cell death, characterized by ROS production, LDs accumulation, chromatin condensation, nuclear envelope separation and DNA damage exposing free 3′OH groups detected by the TUNEL assay indicating the induction of an apoptotic mechanism. This apoptotic induction is specific for some non-albicans Candida species, but not *C. albicans* and *C. neoformans* (**Figure 9**). These results, indicate the existence of diverse mechanisms of microbial interaction between *S. aureus* and different yeast species. Our findings correlate with recent *in vivo* research that demonstrate better survival of mice co-infected with *C. glabrata–S. aureus* that the mice infected with *C. albicans–S. aureus* (Lilly et al., 2018). The production of bacteriocins by *S. aureus* has been reported (Hammami et al., 2013), as thermo-stable peptides (Netz et al., 2001, 2002; Carlin-Fagundes et al., 2016) with activities toward several pathogenic bacterial species (Nascimento et al., 2006; Hammami et al., 2013). The activity we detect in the CFBS, is heat-resistant and it was conserved after filtering the CFBS trough a 3 kDa membrane (not shown) suggesting the presence of an anti-microbial peptide. Further research must be done to identified the exact nature of this compound produced by *S. aureus* capable to induce apoptosis in *C. glabrata*.

## Conclusion

To our knowledge this is, the first report showing an antagonistic behavior produced by *S. aureus* toward *C. glabrata* on *C. glabrata- S. aureus* mixed biofilm, co-culture of *C. glabrata–S. aureus* planktonic cells, and *C. glabrata* in presence with CFBS. The bacterium *S. aureus* and their CFBS inhibit *C. glabrata* growth and actively kill the cells by an apoptotic mechanism. The next steps in our research is to purify and identified the molecule responsible for the antagonistic relationship in the CFBS, after that, if it approves the non-lethality standards in mammalian cells it could probably function as a therapeutic alternative for*C. glabrata* because it shows an acquire resistance to azoles and a high prevalence.

## Availability of Data and Materials

All data generated or analyzed during this study are included in this published article, and its supplementary information files.

## Author Contributions

OC-M participated on standardization of biofilms, carried out SEM and CLSM studies, also performed the statistical analysis, and helped to draft the manuscript. IC-A performed TEM studies of biofilms, the viability assay, and helped to draft the manuscript. CH-R participated in the analysis of results and the drafting of the manuscript. BG-P participated in CLSM studies and in the analysis of results. MM-R participated in the analysis of results and the drafting of the manuscript. AR-T coordinated the design of the study, participated in the analysis of the results, and drafted the manuscript. All authors read and approved the final manuscript.

## Conflict of Interest Statement

The authors declare that the research was conducted in the absence of any commercial or financial relationships that could be construed as a potential conflict of interest.

## Abbreviations

BHI: brain heart infusion medium
CFBS: cell-free bacterial supernatant
CFU: colony forming units
CLSM: confocal laser scanning microscopy
DCFH-DA: 2’,7’-dichlorofluorescin diacetate
DMSO: dimethyl sulfoxide
ECM: extra cellular matrix
EPS: exopolymeric substance
FUN^®^-1: fluorescent vital dye FUN^®^-1(2-chloro-4-[2,3-dihydro-3-methyl-{benzo-1,3-thiazol-2-yl}- methylidene]-1-phenylquinolinium iodide)
LDs: lipid droplets
MRSA: methicillin-resistance *S. aureus*
MTT: tetrazolium salt 3-(4,5-dimethyl-2-thiazolyl)-2,5-diphenyl-2H-tetrazolium bromide
OD: optical density
PBS: phosphate buffered saline
PI: propidium iodide
PQs: phototoxic perylenequinones
ROS: reactive oxygen species
RPMI: Roswell park memorial institute medium
SEM: scanning electron microscopy
SEs: sterol esters
TAGs: triacylglycerols
TEM: transmission electron microscopy
YPD, yeast extract, peptone: dextrose medium.
