# Supplementary material for: Antagonistic Interaction of Staphylococcus aureus Toward Candida glabrata During in vitro Biofilm Formation Is Caused by an Apoptotic Mechanism
Source: Front Microbiol. 2018 Aug 30;9:2031. doi: 10.3389/fmicb.2018.02031 (PMC6125415; doi:10.3389/fmicb.2018.02031)
Supplement: Supplementary file 1 [file Image_1.PDF]

Supporting Information

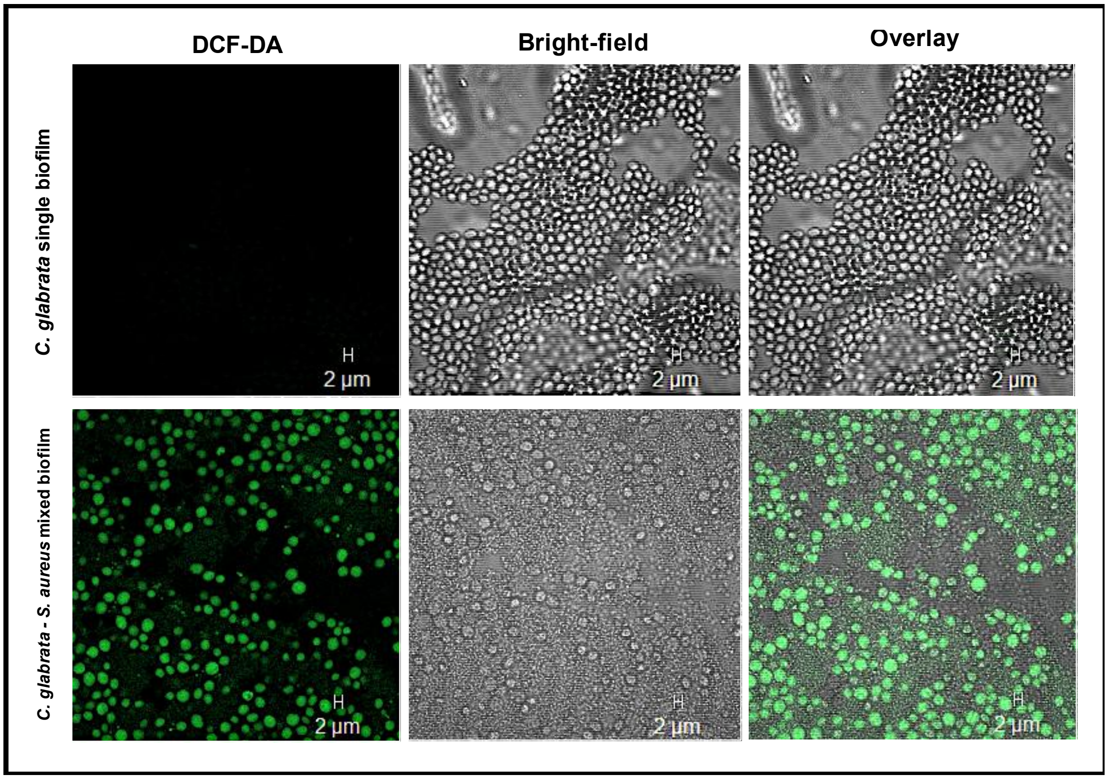

S1 Fig. ROS detection in *C. glabrata* single biofilms and *C. glabrata-S. aureus* mixed biofilms by CLSM.

ROS detection in *C. glabrata* single biofilms and *C. glabrata-S. aureus* mixed biofilms. ROS accumulation was revealed by incubation with 2,7-dichlorodihydrofluorescein diacetate (DCFH-DA) reagent for 24 h at 37ºC. ROS-positive cells were identified by green fluorescence only in *C. glabrata-S. aureus* mixed biofilms. *C. glabrata* single biofilms did not exhibit any fluorescence.
